# Welcome to ONO

**DOI:** 10.1097/ONO.0000000000000002

**Published:** 2021-09-22

**Authors:** Michael E. Hoffer, Elizabeth H. Toh

**Affiliations:** 1 Professor of Otolaryngology and Neurological Surgery, University of Miami, Miller School of Medicine, Miami, Florida; 2 Clinical Professor of Otolaryngology, Boston University School of Medicine, Lahey Hospital & Medical Center, Burlington, Massachusetts.

Another New Journal?

The American Otological Society and American Neurotology Society are proud to bring to you a new addition to the *Otology & Neurotology* family – *Otology & Neurotology Open*. *Otology & Neurotology Open (ONO*) is an online only, gold open access journal created to expand the breadth of published research within the interdisciplinary fields of otology, neurotology, cranial base surgery, and neuroengineering and to disseminate this information freely to the world. We are often asked why we need another new journal? Our response is that the field of otology and neurotology continues to grow warranting a new vehicle to publish high-quality, peer-reviewed research that is globally and freely accessible.

Open access journals represent an important evolution in the world of scientific media. Open access publishing is based on the premise that scientific advances should be globally accessible, not be restricted to only those who have the privilege of access to a medical library that can afford to subscribe to sets of scientific journals. By broadly disseminating important scientific advances without a paywall, the worldwide community of otology and neurotology will benefit from new discoveries and will have the opportunity to participate in developing solutions for the future. The amount of medical knowledge development in our field is exploding and it is critical to provide a fully refereed and rigorously peer-reviewed vehicle to disseminate these developments as soon as possible to everyone who can benefit from these advances. As an online only, open access journal, *ONO* will facilitate knowledge exchange without page limitations. While focusing on high-quality, high-impact research, *ONO* is not subject to any restrictions on the volume of otologic and neurotologic research published. The journal can publish all color illustrations, welcomes the submission of multimedia files, and offers authors the opportunity to publish raw data as supplemental material which can be published with the article.

As Editor-in-Chief and Deputy Editor-in-Chief, we welcome you to explore this important new publication in our field. We have an innovative group of associate editors and a distinguished international editorial board, and tremendous support from our sponsoring societies, our publisher Wolters Kluwer, and our colleagues at *Otology & Neurotology.* As a group, we believe that this new journal is a vital advance in the field of otology and neurotology and for the patients whom we serve all over the world. Please join us in making *ONO* not just another new medical journal but the medical journal of the future. Show that your research impacts everyone in the world—support legitimate open access publication.

*ONO* welcomes global submissions. Members of the American Otological Society and the American Neurotology Society are offered discounted article processing charges (APCs). Additionally, the journal is participating in the Research4Life whereby APCs are fully or partially waived for authors in developing countries.

*ONO* publishes articles continuously on the *ONO* website (www.onojournal.com), striving to publish within a month of acceptance by the Editors. Articles will then be compiled into quarterly issues. In this inaugural issue, we urge you to explore our new publishing platform and to discover a brand-new experience in research literature in the field of otology and neurotology.

Thank you for your support of *Otology & Neurotology Open*.

